# Supplementary material for: Genetic Determinants of Telomere Length in African American Youth
Source: Sci Rep. 2018 Sep 5;8:13265. doi: 10.1038/s41598-018-31238-3 (PMC6125592; doi:10.1038/s41598-018-31238-3)
Supplement: Supplementary file 1 — Supplementary Information [file 41598_2018_31238_MOESM1_ESM.pdf]

Supplementary Information

Genetic Determinants of Telomere Length in African American Youth

Andrew M. Zeiger[1,2,+], Marquitta J. White[1+,*], Celeste Eng[1], Sam S. Oh[1], Jonathan Witonsky[1,3], Pagé C. Goddard[1], Maria G. Contreras[1,4,5], Jennifer R. Elhawary[1], Donglei Hu[1], Angel C.Y. Mak[1], Eunice Y. Lee[1], Kevin L. Keys[1], Lesly-Anne Samedy[1,6], Oona Risse-Adams[1,7], Joaquín Magaña[1], Scott Huntsman[1], Sandra Salazar[1], Adam Davis[8], Kelley Meade[8], Emerita Brigino-Buenaventura[9], Michael A. LeNoir[10], Harold J. Farber[11], Kirsten Bibbins-Domingo[1], Luisa N. Borrell[12], Esteban G. Burchard[1,6]

[1] Department of Medicine, University of California, San Francisco, CA, USA.

[2] Department of Biology, University of Washington, Seattle, WA, USA.

[3] Department of Pediatrics, University of California, San Francisco, CA, USA.

[4] SF BUILD, San Francisco State University, San Francisco, CA, USA.

[5] MARC, San Francisco State University, San Francisco, CA, USA.

[6] Department of Bioengineering and Therapeutic Sciences, University of California, San Francisco, CA, USA.

[7] Lowell Science Research Program, Lowell High School, San Francisco, CA, USA.

[8] Children's Hospital and Research Center Oakland, Oakland, CA, USA.

[9] Department of Allergy and Immunology, Kaiser Permanente–Vallejo Medical Center, Vallejo, CA, USA.

[10] Bay Area Pediatrics, Oakland, CA, USA.

[11] Department of Pediatrics, Section of Pulmonology, Baylor College of Medicine and Texas Children's Hospital, Houston, TX, USA.

[12] Department of Epidemiology & Biostatistics, Graduate School of Public Health & Health Policy, City University of New York, New York, NY, USA.

+ Authors contributed equally to this work.

* Corresponding Author:

Marquitta J. White
Department of Medicine, Lung Biology Center
UCSF, Rock Hall Box 2911, 1550 4th St Rm 584B, San Francisco, CA 94158 USA
Telephone: 415.514.9931
Fax: 415.514.4365
Email: marquitta.white@ucsf.edu

Supplementary Figure S1. Association between log-transformed TL and African ancestry in healthy African American children and adolescents in SAGE: San Francisco Bay Area, 2006-2015

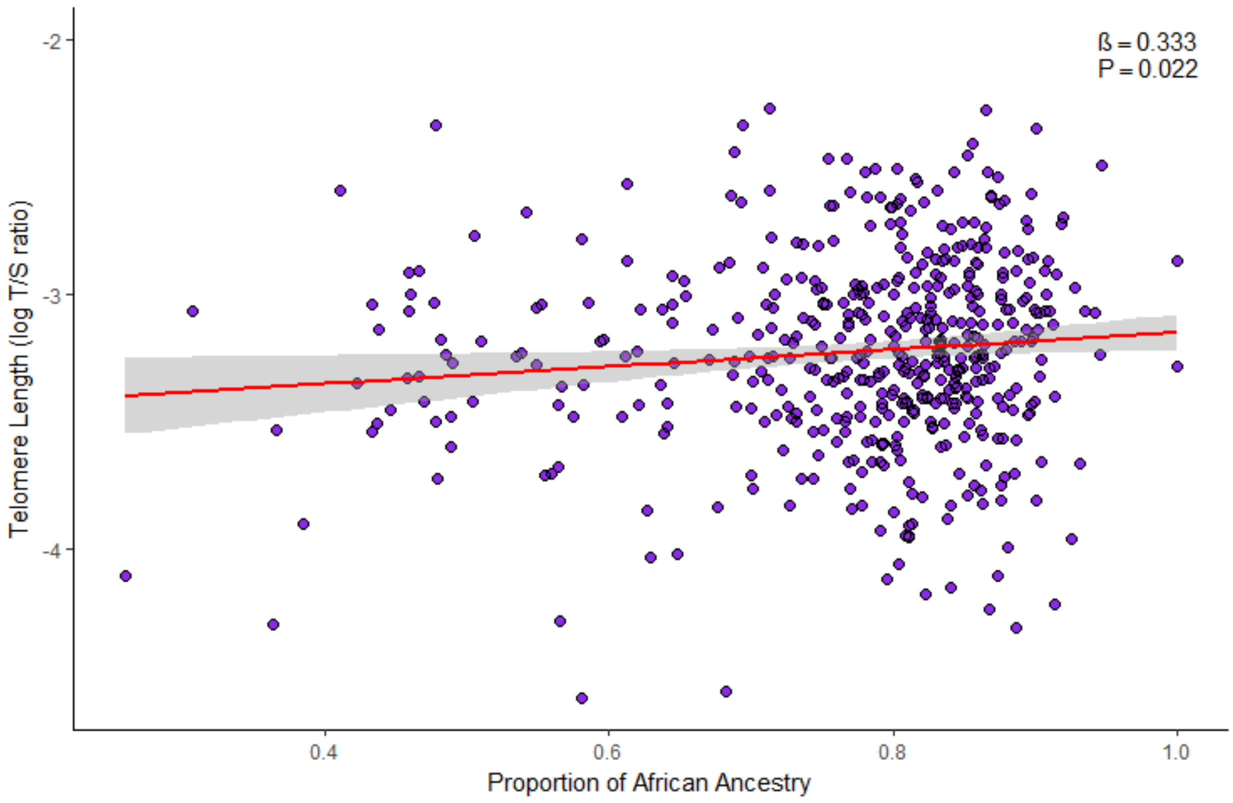

Supplementary Table S1. Associations between covariates and log-transformed TL adjusted for sex in healthy African American children and adolescents in SAGE: San Francisco Bay Area, 2006-2015

Variable	ß (95% CI)	P
Age	-3.07E-3 (-1.27E-2, 6.59E-3)	0.533
Sex[1]	-2.59E-2 (-9.42E-2, 4.24E-2)[a]	0.457
African ancestry	3.33E-1 (4.93E-2, 6.17E-1)	0.022
Maternal education[2]	1.89E-2 (-5.13E-2, 8.91E-2)	0.597
Insurance type[3]	1.29E-1 (6.16E-2, 1.96E-1)	1.84E-4

[a]Association not adjusted for sex.

[1]Reference group = female

[2]Reference group ≤ high school graduate

[3]Reference group = private insurance

Supplementary Table S2: Significant and suggestive associations between common variants and log-transformed TL in healthy African American children and adolescents (n=492) in SAGE: San Francisco Bay Area, 2006-2015

Chr.	Start	Nearest Gene	SNP	Effect Allele (EA)	Minor Allele Frequency	ß (95% CI)	P
1	175463789	*TNR*	rs859393	A	0.058	-0.249 (-0.351, -0.147)	2.24E-06
	175475794		rs859383	C	0.057	-0.246 (-0.346, -0.145)	2.09E-06
3	6081685	*GRM7*	rs73123510	C	0.061	-0.242 (-0.339, -0.146)	1.16E-06
5	95553696	*TTC37*	rs4305653	C	0.142	0.167 (0.010, 0.235)	1.81E-06
	95883847	*ELL2*	rs10055259	A	0.073	-0.193 (-0.268, -0.118)	6.88E-07
	95885405		rs2350242	G	0.071	-0.189 (-0.266, -0.112)	1.83E-06
	165809434	*LINC01947*	rs116511119	G	0.051	0.249 (0147, 0.351)	2.27E-06
6	2739724	*MYLK4*	rs11751430	C	0.091	0.193 (0.114, 0.271)	2.27E-06
11	127499664	*LOC101929497*	rs356273	T	0.276	0.124 (0.074, 0.175)	1.68E-06
13	99094367	*DOCK9-AS2*	rs71437964	T	0.294	0.133 (0.084, 0.183)	2.17E-07
14	37034979	*SLC25A21*	rs116205966	T	0.050	-0.268 (-0.375, -0.161)	1.26E-06
	37035012		rs77290882	A	0.050	-0.268 (-0.375, -0.161)	1.26E-06
	37038801		rs80232634	G	0.050	-0.268 (-0.375, -0.161)	1.26E-06
	42798544	*LRFN5*	rs1351794	C	0.229	0.144 (0.088, 0.200)	7.30E-07
	**42805905**		**rs1483898** [a]	**A**	**0.236**	**0.148 [0.094, 0.200]**	**7.86E-08**
	42807937		rs1843055	C	0.201	0.143 (0.086, 0.201)	1.28E-06
18	20730712	*ROCK1*	rs9675924	A	0.129	-0.171 (-0.241, -0.101)	2.27E-06
20	25275621	*PYGB*	rs73598373	C	0.060	-0.222 (-0.311, -0.133)	1.40E-06
	25337214	*ABHD12*	rs73614590	C	0.066	-0.222 (-0.313, -0.132)	1.98E-06
	25340777		rs73614591	A	0.066	-0.222 (-0.313, -0.132)	1.98E-06
	25355999		rs66501707	A	0.069	-0.226 (-0.317, -0.135)	1.48E-06
	25360402		rs7273525	A	0.069	-0.226 (-0.317, -0.135)	1.48E-06
	25365904		rs73614594	T	0.070	-0.226 (-0.317, -0.135)	1.48E-06
	25384973		rs73331863	A	0.063	-0.226 (-0.317, -0.135)	1.48E-06
	25388258		rs73331878	A	0.063	-0.226 (-0.317, -0.135)	1.48E-06
	25398430		rs73333513	C	0.063	-0.226 (-0.317, -0.135)	1.48E-06
	25430516	*GINS1*	rs7260843	T	0.063	-0.226 (-0.317, -0.135)	1.48E-06
	25440370		rs67679582	G	0.063	-0.231 (-0.322, -0.140)	1.02E-06
	25462472	*NINL*	rs66467007	A	0.062	-0.231 (-0.322, -0.140)	1.02E-06
	25544115		rs73337355	G	0.065	-0.224 (-0.315, -0.133)	1.86E-06
	25558625		rs112449476	T	0.065	-0.224 (-0.315, -0.133)	1.86E-06
	25558791		rs8126111	C	0.065	-0.224 (-0.315, -0.133)	1.86E-06
	25562758		rs8120972	A	0.065	-0.224 (-0.315, -0.133)	1.86E-06
	25563834		rs111469055	T	0.065	-0.224 (-0.315, -0.133)	1.86E-06
	25576983		rs73339335	A	0.063	-0.222 (-0.312, -0.131)	2.14E-06
	25592209		rs7261537	C	0.064	-0.222 (-0.312, -0.131)	2.14E-06
	25601164	*NANP*	rs79132386	C	0.064	-0.222 (-0.312, -0.131)	2.14E-06
	25605527		rs16987934	G	0.064	-0.222 (-0.312, -0.131)	2.14E-06
	25611085		rs73341305	G	0.064	-0.222 (-0.312, -0.131)	2.14E-06
	25619492		rs73341328	G	0.064	-0.222 (-0.312, -0.131)	2.14E-06
	25625588	*ZNF337-AS1*	rs79979640	C	0.065	-0.222 (-0.312, -0.131)	2.14E-06
	25647025		rs80105553	G	0.066	-0.231 (-0.322, -0.140)	8.83E-07

[a]Variant reaches genome-wide significance threshold.

Determination of Relative Telomere Length (TL)

DNA was isolated from whole blood (peripheral blood mononuclear cells) collected from 1,713 SAGE participants (1027 cases and 686 controls) using the Wizard® Genomic DNA Purification Kits (Promega, Fitchburg, WI). Quality and quantity of the extracted DNA were determined using the NanoDrop® ND-1000 spectrophotometer (Thermo Scientific). Absorbance ratios (260/280) were used to assess the quality of DNA. Absorbance ratios for samples were between 1.8 and 2.0.

Relative telomere length for each sample was determined according to the quantitative real time PCR (qPCR) protocol first presented by Cawthon *et al.* with modifications published by O'Callaghan *et al.*[1,2]. Two uniplex qPCR reactions were performed for each sample; one for the telomeric region (T) and the other for single-copy gene (S, *36B4*). Samples were assayed in triplicate for both the T and S amplicons within the same assay. In brief, each 20 µL qPCR reaction master mix contained: 20 ng DNA, 1×AmpliTaq Gold 360 Master Mix (Applied Biosystems, Foster City, CA), 10uM forward and reverse primers (Integrated DNA Technologies) for either T or S, 1X SybrGreen. Cycling conditions (for both T and S amplicons) were: 10 min at 95°C, followed by 40 cycles of 95°C, for 15 s and 60°C for 1 min. Each qPCR plate contained a reference DNA, a no template negative control, and T and S standard curve, as described by O'Callaghan *et al.*[2]. The standard curves were used to evaluate efficiency for both telomere and single copy gene assays. Average amplification efficiency across plates was ≥ 90% for both assays. DNA from the commercially available 1301 lymphoblastic cell line (ECACC 01051619) served as the control DNA for all plates. A description of the oligomers and standards used for the TL assay is provided in Supplementary Table S3, below.

Supplementary Table S3. Oligomers used for TL assay

	**Oligomer Name**	**Oligomer sequence (5'-3')**
**Standards**	Telomere (T)	$(TTAGGG)_{14}$
	36B4 (S)	CAGCAAGTGGGAAGGTGTAATCCGTCTCCACAGACAAGGCCAGGACTCGTTTG
**PCR Primers**	TeloF	CGGTTTGTTTGGGTTTGGGTTTGGGTTTGGG TTTGGGTT
	TeloR	GGCTTGCCTTACCCTTACCCTTACCC TTACCCTTACCCT
	36B4F	CAGCAAGTGGGAAGGTGTAATCC
	36B4R	CCCATTCTATCATCAACGGGTACAA

Cycle threshold (C_t) values for telomere (T) and single-copy gene (S) were determined using the ABI 7900HT Fast Real-Time PCR System with SDS Ver. 2.4 software (Applied Biosystems, Foster City, CA, USA). Each qPCR experiment was run in triplicate and accepted only if the standard deviation of the C_t values were < 1 C_t; accepted C_t values were then averaged for each sample. After assessing C_t values using this metric, 114 samples were removed from further analysis. After quality control procedures, TL was computed for a total of 596 healthy controls from the Study of African Americans, Asthma, Genes and Environments (SAGE). Relative TL was calculated using the $2^{-\Delta\Delta Ct}$ formula published by Cawthon *et al.*[1].

References:

1. Cawthon, R. M. Telomere measurement by quantitative PCR. *Nucleic Acids Res.* **30,** e47 (2002).
2. O'Callaghan, N. J. & Fenech, M. A quantitative PCR method for measuring absolute telomere length. *Biol. Proced. Online* **13,** 3 (2011).
